# Biomechanical Effects of Seizures on Cerebral Dynamics and Brain Stress

**DOI:** 10.3390/brainsci14040323

**Published:** 2024-03-27

**Authors:** Molly Bekbolatova, Jonathan Mayer, Rejath Jose, Faiz Syed, Gregory Kurgansky, Paramvir Singh, Rachel Pao, Honey Zaw, Timothy Devine, Rosalyn Chan-Akeley, Milan Toma

**Affiliations:** 1 Department of Osteopathic Manipulative Medicine, College of Osteopathic Medicine, New York Institute of Technology, Old Westbury, NY 11568, USA; mbekbola@nyit.edu (M.B.); jmayer03@nyit.edu (J.M.); rjose02@nyit.edu (R.J.); fsyed09@nyit.edu (F.S.); gkurgans@nyit.edu (G.K.); psingh64@nyit.edu (P.S.); 2 NewYork-Presbyterian Queens Hospital, New York City, NY 11355, USA; dxy9002@nyp.org; 3 Icahn School of Medicine at Mount Sinai, 1428 Madison Avenue, Atran Berg Building, 8th Floor, New York City, NY 10029, USA; honey.zaw@mssm.edu; 4 The Ferrara Center for Patient Safety and Clinical Simulation, Department of Osteopathic Manipulative Medicine, College of Osteopathic Medicine, New York Institute of Technology, Old Westbury, NY 11568, USA; tdevine@nyit.edu; 5 Pfizer Inc., 235 E 42nd St, New York City, NY 10017, USA; rosalyn.chan@aya.yale.edu

**Keywords:** seizure, simulations, computational, brain, CSF

## Abstract

Epilepsy is one of the most common neurological disorders globally, affecting about 50 million people, with nearly 80% of those affected residing in low- and middle-income countries. It is characterized by recurrent seizures that result from abnormal electrical brain activity, with seizures varying widely in manifestation. The exploration of the biomechanical effects that seizures have on brain dynamics and stress levels is relevant for the development of more effective treatments and protective strategies. This study uses a blend of experimental data and computational simulations to assess the brain’s physical response during seizures, particularly focusing on the behavior of cerebrospinal fluid and the resulting mechanical stresses on different brain regions. Notable findings show increases in stress, predominantly in the posterior gyri and brainstem, during seizures and an evidence of brain displacement relative to the skull. These observations suggest a dynamic and complex interaction between the brain and skull, with maximum shear stress regions demonstrating the limited yet essential protective role of the CSF. By providing a deeper understanding of the mechanical changes occurring during seizures, this research supports the goal of advancing diagnostic tools, informing more targeted treatment interventions, and guiding the creation of customized therapeutic strategies to enhance neurological care and protect against the adverse effects of seizures.

## 1. Introduction

Seizures can be a distressing and worrisome experience for both the patient and their caregivers. They are one of the oldest recognized medical conditions in the world. Written records dating as far back as 4000 BCE document the existence of epilepsy [1]. In the United States, approximately 1.2% of the population had active epilepsy in 2015, which translates to about 3.4 million people nationwide. This includes 3 million adults and 470,000 children [2]. The risk of premature death in people with epilepsy is up to three times higher than in the general population. A significant proportion of epilepsy-related deaths, especially in low- and middle-income countries and rural areas, are potentially preventable, such as those caused by falls, drowning, burns, and prolonged seizures [3]. In a cohort study, it was found that over 70% of patients developed lasting remission from seizures. However, the mortality rate in the long term was still twice that of the general population [4]. In recent years, innovative therapies for epilepsy management have emerged, grounded in novel principles, supported by increasing clinical evidence, and showing promise for application in diverse patient populations [5].

A seizure occurs when there is abnormal electrical activity in the brain, leading to convulsions, loss of consciousness, or unusual movements. Parents and guardians need to recognize the signs and symptoms of a seizure, such as blank stares, jerking movements, or sudden unawareness [6]. Although seizures can have different causes like fevers, epilepsy, or head injuries, it is essential to seek medical attention from healthcare professionals for proper diagnosis and management [7]. By providing appropriate care and understanding, people with seizures can lead fulfilling lives with the necessary support to manage their condition effectively. Henceforth, it is imperative to delve into studying this condition to broaden our comprehension so that we may offer adequate care—which forms the core purpose of this paper.

In general, during a seizure, when the body shakes, several intricate physiological changes occur in the brain. These changes are caused by abnormal and synchronized electrical activity within the neural networks of the brain [8]. Seizures involve excessive and disorganized firing of neurons, leading to disruptions in motor function, sensory perception, and cognitive processes [9]. The impact on various regions and functions of the brain during shaking episodes is complex. Unfortunately, clinical studies have shown that refractory seizures occurring early in life can have significant negative effects on cognitive abilities despite treatment with antiepileptic medications [10].

Seizures are complex events marked by a sudden surge of electrical activity in the brain, often characterized by neuronal hyperactivity [11]. This hyperactivity can cause a widespread synchronization of neuron firing, disrupting the normal communication within the brain, which can lead to motor symptoms such as shaking or convulsions [12,13]. One of the areas often activated during these episodes is the motor cortex, the brain’s control center for voluntary movements, which, when overstimulated, can result in involuntary, exaggerated muscle contractions [14]. Additionally, the thalamus, crucial for relaying sensory and motor signals, can experience functional disturbances during seizures, leading to abnormal motor behaviors, including shaking [15]. The basal ganglia, which are involved in motor control and movement coordination, can also be activated during a seizure, further contributing to the lack of motor control and involuntary movements typical of shaking episodes [16]. While the hippocampus does not directly cause shaking, it is critical for memory and spatial navigation and its involvement in seizures can lead to cognitive disturbances [17]. Seizures also disrupt the brain’s typical electrical patterns, creating highly synchronized brain waves that contribute to the physical manifestations [18]. The intense neuronal activity of seizures requires a high consumption of energy, which can change the brain’s metabolism, potentially causing fatigue or confusion afterwards [19]. Moreover, an imbalance in neurotransmitters, the brain’s chemical messengers, can occur during a seizure, contributing to a variety of effects, including those affecting motor function [20]. Consequently, during a seizure with shaking episodes, the brain undergoes a cascade of physiological changes affecting different brain regions and neural networks [21], leading to motor manifestations, altered perception, cognitive effects, and other symptoms associated with seizures [22]. The specific effects vary widely and are heavily dependent on the seizure’s type and location in the brain [23].

A collection of scholarly articles described below delves into the physiological effects of seizures on the brain during episodes of shaking, providing valuable insights into the underlying mechanisms. A study by Blumenfeld et al. used single-photon-emission computed tomography to examine how blood flow patterns in the brain change during generalized tonic–clonic seizures [24]. The results indicated that certain regions of the brain, including the superior medial cerebellum, thalamus, and basal ganglia, showed increased blood flow during seizures. These findings suggest that these areas may play a role in the motor symptoms associated with seizures. In a recent study by Brodovskaya et al., researchers investigated the activation patterns of neurons in the basal ganglia during seizures originating from the frontal lobe and leading to bilateral tonic–clonic movements in rats [25]. The results indicated significant neuronal activation within various regions, including the striatum, globus pallidus externus, subthalamic nucleus, and substantia nigra during these seizures. Moreover, there was a notable preference for D2 dopamine receptor neurons in the indirect pathway of the basal ganglia. These findings contribute to our understanding of how the basal ganglia are involved in motor disturbances associated with seizures. In a study conducted by Canas et al., the focus was on examining the impact of adenosine A2A receptors (A2AR) in hippocampal neurodegeneration caused by convulsions, using an animal model of temporal lobe epilepsy [26]. The results indicated that there was a rapid increase in A2AR levels within glutamatergic synapses, which resulted in synaptic damage, diminished synaptic plasticity, and loss of neurons. These findings propose a potential association between adenosine receptors and the occurrence of seizure-induced neurodegeneration. A study conducted by DeSalvo et al. in 2010 investigated the changes in brain activity during bicuculline-induced tonic–clonic seizures in rats using blood oxygen level-dependent (BOLD) fMRI signals [27]. The results revealed that there were noticeable alterations in the cortex, brainstem, and thalamus with increased BOLD signals, whereas a decrease was observed in the hippocampus. These findings emphasize how tonic–clonic seizures primarily affect specific regions of the brain. Another study investigated how seizures contribute to neuronal damage and cell death after mild traumatic brain injury [28]. By inducing seizures in rats using a low dose of PTZ, researchers observed an increase in the number of apoptotic cells in the TBI+PTZ group. Molecular analysis showed that the mitochondrial and caspase-related apoptotic pathways were activated, possibly as a result of GABAergic disinhibition. These findings suggest that seizures could worsen neuronal loss in different areas of the brain. The Green et al. study conducted research on the effect of convulsions on GABA release by examining brain slices from rats that were exposed to electroconvulsive shock or flurothyl [15]. The findings revealed that convulsions resulted in a decrease in potassium-evoked GABA release across different regions of the brain. These findings offer important information about how inhibitory neurotransmission is influenced during seizure activity. The reviewed scholarly articles collectively shed light on the intricate physiological effects of seizures on the brain during episodes of shaking. These studies enhance our understanding of the neural mechanisms underlying seizure-related motor manifestations, neurodegeneration, and alterations in neurotransmitter release. Such insights have implications for advancing our knowledge of epilepsy and potentially developing targeted interventions for seizure management.

The cerebrospinal fluid (CSF) has a crucial role in protecting the brain. It serves as a cushion or buffer, providing basic mechanical and immunological protection to the brain inside the skull [29]. During seizures, there may be abrupt and severe movements, which, if unbuffered, could lead to brain damage [30]. The CSF helps in dissipating these forces and reducing the risk of brain injury [31]. In the context of this study, smoothed particle hydrodynamics (SPH) in combination with high-order finite element method (FEM) were used to simulate the behavior of CSF during seizures or shaking episodes. SPH can offer a comprehensive understanding of how force is distributed within the CSF and transmitted to the brain, improving our knowledge of how CSF dynamics can affect different areas of the brain during seizures. It is important to note that although CSF provides some level of protection, seizures can still lead to physiological changes and have various impacts on the brain [32]. The specific effects may vary depending on factors such as seizure type, intensity, and location within the brain; however, these aspects were not examined in this study. Overall, this study highlights the role of CSF in mitigating brain damage during seizures and the potential of SPH simulations to provide insights into CSF dynamics and its impact on brain protection.

## 2. Methods

Various simulation methods are utilized to investigate the dynamics of CSF. Two commonly employed techniques include SPH and FEM. SPH is a computational approach often used in fluid dynamics, enabling detailed simulations of complex free surface flows. This method provides valuable insights into the interaction between the brain and CSF under different conditions, making it especially relevant for biomedical research involving intricate fluid–structure interactions (FSI) within the human body [33,34]. On the other hand, FEM offers another popular avenue for simulating CSF dynamics by numerically analyzing structural aspects as well as fluid and air flow phenomena. Thanks to its adaptable nature in dealing with complicated geometries and boundary conditions, this method can effectively simulate CSF movement within real human brains [35]. Computational fluid dynamics (CFD) is a commonly used numerical method. It is a computational method to analyze and simulate the behavior and interaction of fluids with various surfaces or structures, or within themselves. CFD provides detailed insights into the flow patterns, pressure distributions, turbulence, and thermal characteristics of fluid systems. In this context, CFD is instrumental in our understanding of cerebrospinal fluid dynamics within the brain, allowing us to assess the complex interactions between the fluid and the solid tissues during pathological conditions such as seizures. Various techniques are employed depending on the research question at hand. Multiphase models capture different phases within the CSF mixture, while porous media models represent CSF flow through brain tissue as a porous material. The immersed boundary method handles deformable boundaries and complex geometries encountered in brain and CSF motion [36]. In our study, we utilized SPH methodology combined with higher-order FEM to maintain the complexity of brain structures during simulations.

### 2.1. Smoothed Particle Hydrodynamics

**Figure 1:**
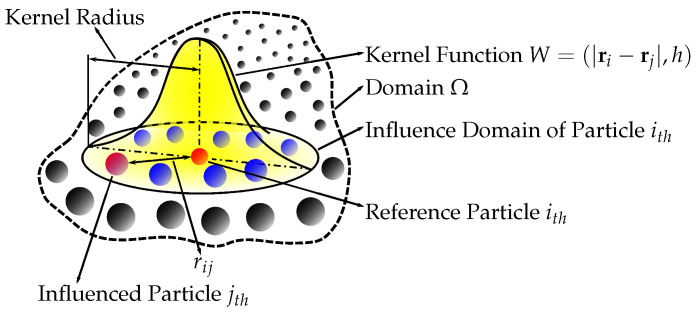
Smoothed-particle hydrodynamics kernel approximation.

Smoothed particle hydrodynamics is a computation technique used in the field of fluid dynamics (Figure 1). This mesh-free method is notable for its capability to simulate complex free surface flows and has found extensive application in various biomedical fields, particularly in the investigation of health conditions associated with the brain. One of the key aspects of using SPH in biomedical research is the ability to model complex FSIs within the human body. In the context of brain injuries, SPH has been instrumental in enhancing our understanding of the interaction between the brain and the cerebrospinal fluid under various loading conditions.

Figure 1 showcases the core concepts of the SPH method, where the key elements are (a) particles illustrated as points or circles scattered within a domain, representing discrete elements of the fluid; (b) the influence domain, which is the region around a reference particle within which other particles exert influence on the reference particle’s properties; (c) a kernel function (W), which is the central element of SPH characterizing how the properties of particles are smoothed over space; (d) the kernel radius (h), which is the range or extent of the kernel function’s influence around a particle; (e) a reference particle (particle $i^{th}$), which is the focal particle, whose properties are being influenced by neighboring particles; (f) influenced particles (particle $j^{th}$), which are the particles contributing to the properties of the reference particle within its influence domain; and (g) $r_{ij}$, which is a vector or line indicating the distance between two particles (*i* and *j*), which plays a role in calculating the influence of particles on each other.

The CSF, present within the subarachnoid space and other cavities inside the brain, plays a crucial role in both protecting the brain from trauma and maintaining its physiological functions. SPH simulations can provide comprehensive insights into the behavior of the CSF during an impact or shaking condition, which might occur during a motor accident or a seizure episode.

Under the conditions of a traumatic brain injury, for instance, the detailed simulation of FSIs by SPH can help investigate the pressure distribution within the CSF and its transmission to the brain. This involves substantial deformation and movement of the brain within the skull, which can cause brain injuries. In the case of seizures and related shaking episodes, the SPH method enables researchers to understand better how the abnormal neuronal activity impacts the CSF dynamics and movement and subsequently affects different regions of the brain, such as the cerebrum, cerebellum, brain stem, and others. By promoting a more vivid depiction of these intricate FSIs, SPH simulations can contribute valuable insights about the underlying mechanisms of brain injuries, which may be pivotal in designing advanced therapies or preventive methods. Thus, SPH serves as an essential tool in biomedical applications, providing an in-depth investigation of complex health conditions and significantly enhancing our understanding of brain injuries and their relationship with CSF dynamics.

### 2.2. Geometrical Model

**Figure 2:**
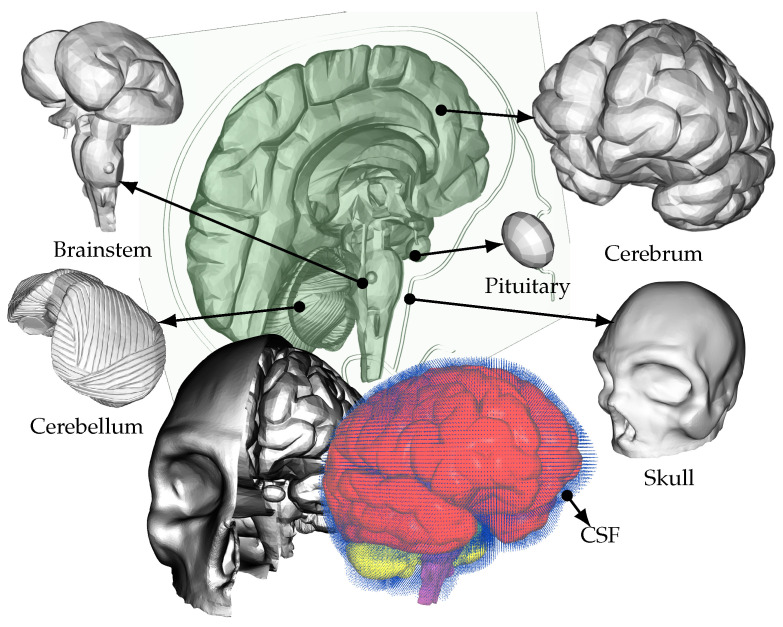
Detailed representation of the comprehensive human brain model showing structures such as the cerebrum, cerebellum, pituitary gland, and brainstem. The skull and the cavities filled with fluid particles, including the subarachnoid space, are also depicted. Half of the skull is selectively removed in the lower-left portion of the image for enhanced visualization.

In this study, a detailed geometrical model is employed to simulate FSI within the brain. Hence, this model takes into account the dynamic relationship between the brain tissue and CSF. The geometrical model used in this study incorporates intricate features found in real brains, including gyri and sulci, which are the characteristic folds and grooves on the brain’s surface (Figure 2). Numerous current models discussed in academic sources opt to streamline particular elements, for instance by omitting realistic anatomical characteristics like sulci and gyri or neglecting the representation of the fluid domain (cerebrospinal fluid) and substituting it with solid elements [37]. In contrast, this model prioritizes precision by maintaining a more intricate level of anatomical detail and utilizing an FSI method.

For the reader’s convenience, we reiterate information on the five distinct anatomical structures utilized in this model, as detailed in our prior publications, e.g., [38]. These structures include the skull, cerebrum, cerebellum, pituitary gland, and brainstem, each having unique material properties. The patient-specific model is based on Digital Imaging and Communications in Medicine (DICOM) images obtained from an online database. However, it should be noted that certain anatomical features are missing from this model, such as the skin, spinal cord, meninges, and arachnoid granulation. In terms of material properties, the skull is assigned rigid characteristics with a density of 1900 kg/$m^{3}$ [39]. On the other hand, studies examining the macroscopic physical properties of the brain indicate that it behaves as a viscoelastic material [40]. Therefore, the cerebrum, cerebellum, pituitary gland, and brainstem are simulated using a nonlinear elastic constitutive material model. The material properties for these structures are based on the literature [41,42,43,44]. The number of tetrahedral elements used to model each structure are as follows: 96,385 for the cerebrum, 40,808 for the cerebellum, 18,634 for the pituitary gland, and 310 for the brainstem. The CSF is modeled with a bulk modulus of 21.9 GPa and a density of 1000 kg/$m^{3}$ [45]. The subarachnoid space between the skull, brain, and other internal cavities like the ventricles is filled with more than 146,000 fluid particles in this model.

Moreover, this model also includes two separate hemispheres, replicating the asymmetry observed in actual brains. By considering the distinct properties and interactions between the left and right hemispheres, the model provides a more comprehensive understanding of the FSI dynamics within the brain. Furthermore, the subarachnoid space, which surrounds the brain, is filled with fluid particles in this model. This includes the ventricles, where the CSF is produced in real brains [46]. By incorporating the fluid particles within the subarachnoid space and ventricles, the model enables the investigation of CSF flow and its impact on the surrounding brain tissue.

It is worth noting that many competing models often simplify the interactions within the brain by considering the CSF as a deformable solid, neglecting the essential FSI. By contrast, this model recognizes the crucial role of FSI in accurately representing the dynamics of the brain. Additionally, other simplified geometrical models used in previous studies lack the real-life complexity of the brain. They often omit the inclusion of gyri, sulci, separate hemispheres, and other anatomical features. In contrast, this model embraces the complexity of the brain’s geometry, ensuring a more realistic representation and a deeper understanding of the FSI within the brain.

Having separate and distinct models for different parts of the brain enables us to prescribe detailed material properties to each region individually. This approach acknowledges the fact that different brain regions possess unique characteristics and behaviors. By assigning specific material properties to each region, such as the gyri, sulci, and separate hemispheres, we can capture the diverse mechanical responses and functionalities exhibited by these areas. For example, certain regions may have different stiffness or viscoelastic properties, reflecting their distinct anatomical composition and physiological functions [47,48]. This level of granularity in material properties allows for more accurate simulations and predictions of brain behavior. It enables researchers and clinicians to study how different regions of the brain respond to external forces, such as trauma or surgery, and how such interactions can influence overall brain function.

Moreover, this approach also opens up possibilities for personalized medicine and treatment planning. By tailoring material properties to specific brain regions, clinicians can simulate and analyze the effects of interventions or therapeutic strategies on different parts of the brain individually. This level of precision can aid in optimizing treatment approaches, minimizing potential risks, and maximizing the desired outcomes for patients. Having separate and distinct brain models with detailed material properties for each region enhances our ability to capture the complexities of brain mechanics. It allows us to study the unique behaviors of different brain regions and provides a foundation for developing personalized treatment approaches in the field of neurology and neurosurgery.

### 2.3. Boundary Conditions

**Figure 3:**
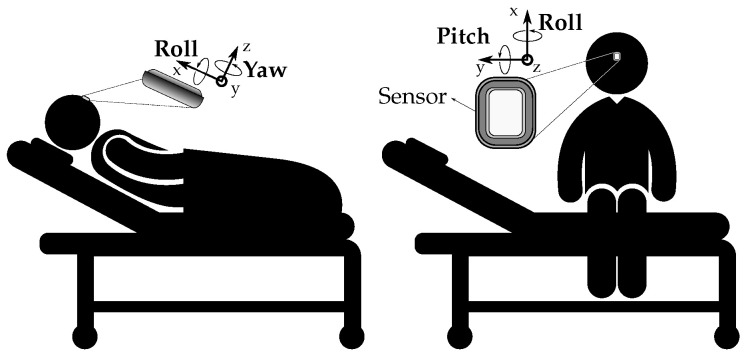
Illustration of pitch, roll, and yaw relative to a patient in both supine and sitting positions.

For this study, we utilized a sophisticated toddler-sized mannequin model that is commonly used in medical colleges and simulation centers. This specific mannequin is frequently employed by students to practice different patient interventions, including the management of seizures. To capture data throughout our experiments, we installed sensors on the mannequin capable of recording velocities, accelerations, and Euler angles over specific time intervals (Figure 3). These recorded values were wirelessly transmitted via Bluetooth to avoid any interference caused by cables that could potentially impact the performance of the mannequin. The captured data can be found in detail in the ‘Results’ section below. Subsequently, we prescribed the acceleration values to the skull in our computational simulations.

The sensors used are called the Movella DOT Sensors (Movella, Inc., Henderson, NV, USA), which are part of a wearable sensor development platform. This platform incorporates a state-of-the-art signal processing and sensor fusion framework that has been optimized for human movement applications. The Movella DOT utilizes Bluetooth low-energy (BLE) technology and comes with a software development kit (SDK). The Movella DOT consists of an inertial measurement unit (IMU) that includes an accelerometer to measure acceleration, a gyroscope to measure rotational speed, and a magnetometer to measure the magnetic field. These components collect individual data, which can be combined through sensor fusion algorithms to calculate the orientation accurately.

Overall, the utilization of the advanced mannequin coupled with the Movella DOT sensors has allowed us to collect accurate and reliable data on motion-related parameters. This combination of advanced technology and wearable sensors offers tremendous potential for further advancements and applications in the field of medical simulation and beyond.

### 2.4. Validation

In a previous study, we employed this geometric model and computational approach to replicate coup and contrecoup brain injuries [38]. To verify the accuracy of this methodology along with the geometrical model, we compared our simulation results to experimental cadaver tests conducted by Nahum et al. [49]. These cadaveric tests involved applying an impact impulse to the frontal lobe of human specimens and measuring the resulting pressure responses in the cerebrospinal fluid for comparison with our computational simulations.

We observed a strong correlation between our simulation results and the cadaver data for the coup response. The contrecoup computational pressure response was slightly elevated compared to the observed values in the cadaver data. However, this difference can be attributed to variations in patient-specific geometries used during the simulation, as contrecoup is a secondary reaction more prone to these variations. Nevertheless, both coup and contrecoup pressure responses from our computational model aligned well with the experimental findings, affirming successful validation efforts.

Additionally, we have performed sensitivity analyses using both broader and coarser meshes, as well as varying the number of fluid particles representing the CSF. Hence, we have considered the potential impact of both the mesh refinement and fluid particle density on the results. Specifically, we have systematically increased and decreased the number of mesh elements and fluid particles by 10%. Upon evaluating the results, we observed notable differences between the broader meshes and the meshes that were utilized to obtain the results presented in this study. However, there was only negligible difference between the meshes (and number of fluid particles) we used and the coarser meshes (and higher number of fluid particles). By performing these sensitivity analyses, we aimed to ensure the robustness and reliability of our findings.

Our investigations diverge from many existing studies in the domain of brain biomechanics, which often utilize purely structural approaches for their simulations. The common practice of approximating cerebrospinal fluid with structural elements neglects the FSI that is crucial for understanding the complex dynamics between the CSF and the brain’s solid tissues [50]. The unique implementation of FSI methods in our study allows us to capture this interaction more realistically, providing a more accurate depiction of the mechanical stress exerted on the brain tissue during seizure episodes. Furthermore, unlike the simplified geometric models that are frequently employed in the field [50,51], which often treat the brain as a uniform mass, our advanced model incorporates the distinct anatomical features of the brain, including gyri and sulci, and represents the brain as two individual hemispheres. This higher level of anatomical detail enables a more nuanced analysis of the biomechanical response of different brain regions during seizures. While direct comparisons with other studies are challenging due to these methodological advancements, our results indicate that incorporating detailed FSI models should be a priority for future research to enhance our understanding of seizure mechanics and their implications for brain health.

## 3. Results

**Figure 4:**
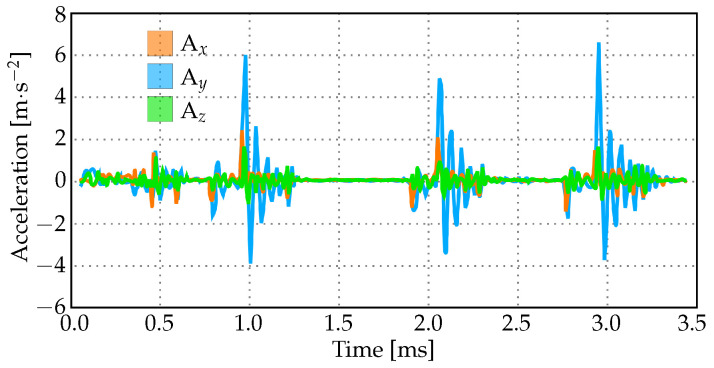
Acceleration values (in *x*, *y*, and *z* directions) measured with sensors placed on a simulation mannequin with the seizure function.

The acceleration measurements during a simulated seizure are depicted in the graph shown in Figure 4. The recorded acceleration values vary between approximately −4 m·$s^{-2}$ and +6 m·$s^{-2}$ in the *x*, *y*, and *z* directions. Within this range of values, there are three distinct peaks observed at intervals of around one second. It is clear from the data that the highest magnitudes of acceleration occur in the *y* direction when compared to the *x* and *z* components. This indicates that during the seizure, there was a significant ‘pitch’ movement, as illustrated in Figure 3.

**Figure 5:**
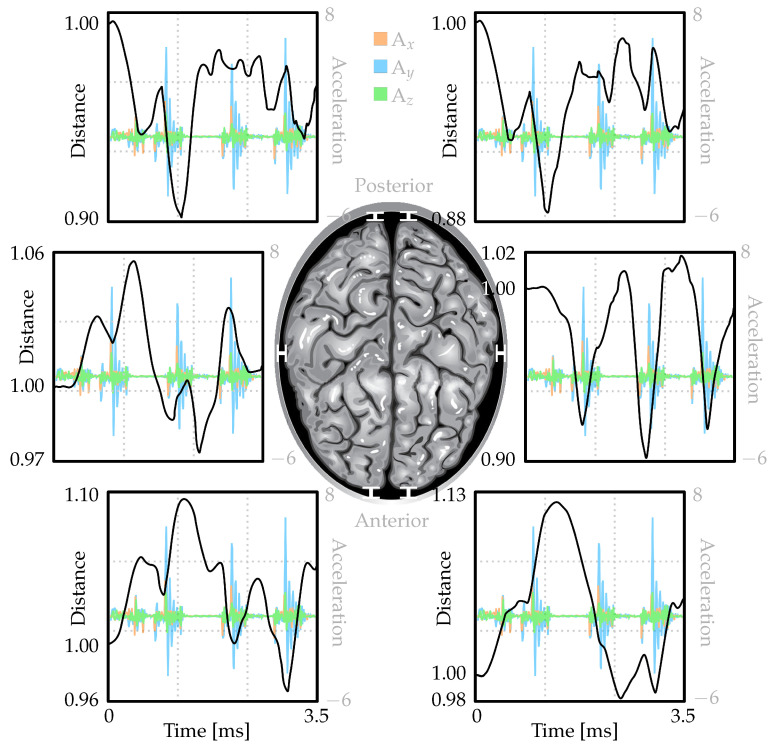
Scaled distance values (scaled by the respective distances in the initial state, i.e., before the seizure) between the skull and brain in six different locations. Hence, when the black line is above one, it indicates an increase in distance between the brain and skull, while a value below one denotes a decrease in distance compared to the initial value.

Figure 5 illustrates the movement of the brain relative to the skull during head accelerations commonly observed in seizures (see Figure 4). The graph presents scaled distances between the skull and brain at six different locations over time. These measurements have been adjusted based on their initial values before the seizure (T = 0 ms). When the values exceed one, it signifies an increase in separation between the brain and skull, whereas a value below one denotes a decrease in distance. Despite experiencing oscillatory acceleration patterns (also depicted in the graphs), the fluctuations in brain–skull distances are relatively smaller due to CSF’s cushioning effect.

Based on the analysis of Figure 5, several conclusions can be drawn. For example, in the posterior region, all scaled distance values over time are less than one (i.e., decreasing), while most values in the anterior region exceed one (i.e., increasing). This suggests that the brain moves backward relative to the skull. Additionally, both hemispheres show similar curve patterns in the posterior section, suggesting that they are equally influenced by the prescribed seizure-induced accelerations of the head. Furthermore, an overall leftward shift of the brain with respect to the skull can be deduced from the increase in distance measured in the right hemisphere and decrease in distance measured in the left hemisphere. Moreover, while both hemispheres display similar curve patterns in the posterior region, there is no such similarity noticed in the anterior section, signifying distinct deformations between the left and right hemispheres at this particular location.

Understanding the distribution and magnitude of contact pressure and stress on different regions of the brain during acceleration is crucial for assessing the potential impact on brain health and injury prevention strategies. In this study, different aspects of the brain’s response to the simulated seizure are demonstrated using the following three calculated results. (1) Contact Pressure: This is the amount of force per unit area exerted at the interface between two contacting bodies. In the context of this study, this refers to the pressure that is exerted where the cerebrospinal fluid comes into contact with the brain. (2) Effective Stress: In the context of poroelasticity, effective stress is the stress that is borne by the solid phase of a porous material, which in this study is the brain tissue. It is the difference between the total stress and the pore pressure, taking into account the influence of the fluid pressures (from CSF) in the pores of the material. (3) Maximum Shear Stress: Shear stress is the measure of the force per unit area that is exerted parallel or tangent to the face of an object or a material. The maximum shear stress is the highest value of this stress that a material experiences. In this study, it represents the greatest shear stress exerted on the brain due to interactions between the CSF and the brain during the simulated seizure event.

**Figure 6:**
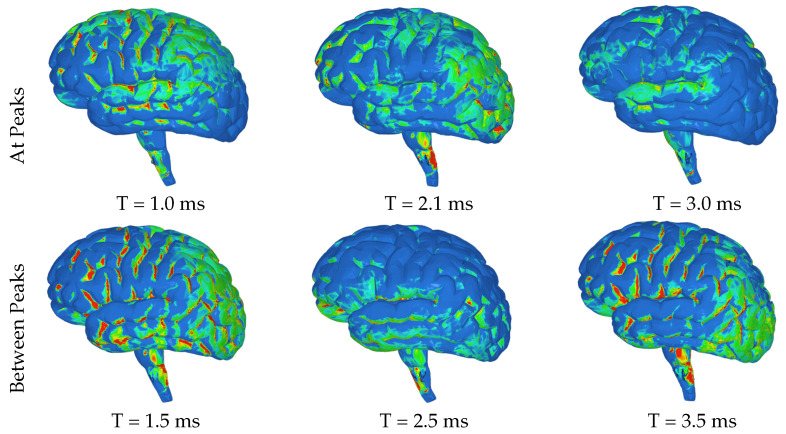
Images of left hemisphere with color map denoting the contact pressure between the fluid and solid domains ranging from 0 MPa (blue) to 4 MPa (red).

**Figure 7:**
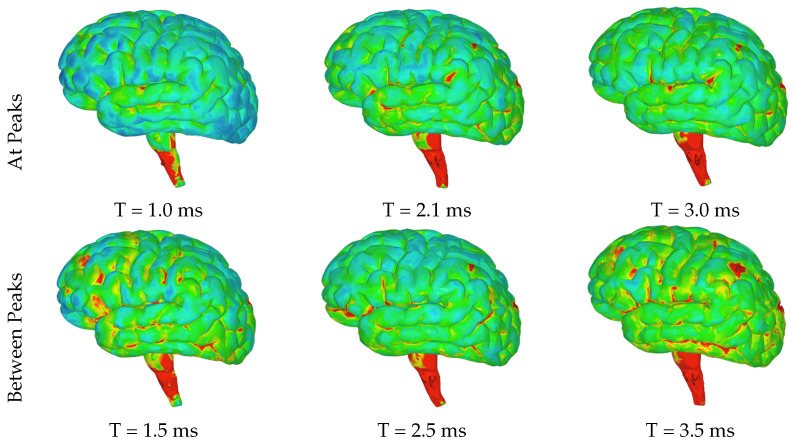
Images of left hemisphere with color map denoting the effective stress exerted on the brain due to the FSI between the CSF and brain ranging from 0 MPa (blue) to 3 MPa (red).

**Figure 8:**
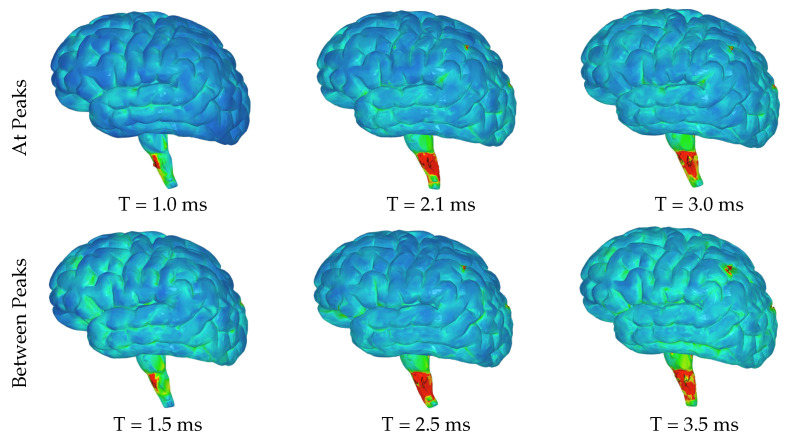
Images of left hemisphere with color map denoting the maximum shear stress exerted on the brain due to the FSI between the CSF and brain ranging from 0 MPa (blue) to 5 MPa (red).

**Figure 9:**
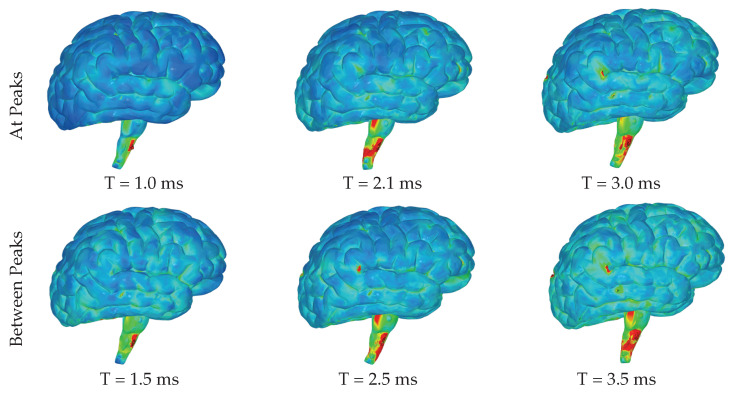
Images of right hemisphere with color map denoting the maximum shear stress exerted on the brain due to the FSI between the CSF and brain ranging from 0 MPa (blue) to 5 MPa (red).

The highest levels of contact pressure resulting from the movement of fluid particles around the brain, as shown in Figure 6, are primarily observed on the posterior sides of the gyri and brainstem following both the initial and third acceleration peaks. Similarly, according to the color map indicating effective stress values, significant stress is exerted on different areas of the brain after both accelerations; however, notably high-stress values occur specifically within the brainstem and the posterior regions of the gyri (Figure 7). Again mirroring trends seen with contact pressure and effective stress, when calculating maximum shear stress values exerted on the brain, elevated levels can be observed primarily within the brainstem (Figure 8 and Figure 9). Notably, these elevated values seem to be more pronounced on the left side of the brainstem (Figure 8) than on the right side (Figure 9), indicating an asymmetry in exerted stress on the brain.

To reiterate, Figure 5 presents a detailed graphical representation of the fluctuations in distances between the brain and the skull at six different positions over a span of time during a simulated seizure. These distances have been scaled against their initial values before the seizure (T = 0 ms). A value of one indicates no change in the distance, while a value greater than one signifies an increase in separation, and a value less than one indicates a decrease in separation. In the posterior region of the brain, all scaled distance values over time are less than one, indicating a decreasing distance. In the anterior region, most values exceed one, signifying an increasing distance. This suggests that the brain tends to move backward relative to the skull during the simulated seizure. Both hemispheres show similar patterns in the posterior region, which is an indication that they are equally affected by the head accelerations caused by the seizure. However, in the anterior region, no such similarity is observed, which implies distinct deformations between the left and right hemispheres at this particular location. Moreover, the graph also suggests an overall leftward shift of the brain relative to the skull, as indicated by the increase in distance measured in the right hemisphere and the decrease in distance measured in the left hemisphere.

Figure 6, Figure 7, Figure 8 and Figure 9 in this study present different aspects of the brain’s behavior during simulated seizure episodes. Figure 6 illustrates the contact pressure between the fluid and solid domains of the brain during seizure episodes, showing this pressure in a range from 0 MPa (blue) to 4 MPa (red). The highest contact pressures occur primarily on the posterior sides of the gyri and brainstem. Figure 7 presents the effective stress exerted on the brain, which is a result of the FSI between the cerebrospinal fluid and the brain. The stress values range from 0 MPa (blue) to 3 MPa (red), with the highest levels observed in the brainstem and the posterior regions of the gyri. Figure 8 shows the maximum shear stress exerted on the left hemisphere of the brain. The color map illustrates this shear stress from 0 MPa (blue) to 5 MPa (red). Notably, the figure highlights higher stress values primarily within the brainstem. Meanwhile, Figure 9 displays the same parameter (maximum shear stress) interpreted for the right hemisphere of the brain. The stress levels show a similar distribution to the left hemisphere except for the brainstem, where lower levels are observed. These figures collectively suggest that during simulated seizures, the brain experiences significant contact pressure, effective stress, and shear stress, predominantly in the brainstem and posterior regions of the gyri.

## 4. Discussion

The collective insights from recent studies demonstrate the promise of innovative and complementary research in developing more precise and effective management approaches for epilepsy. Recent advancements in seizure management highlight the importance of a multidisciplinary approach, integrating findings from various research studies. According to Nurimanov et al., targeted embolization has shown effectiveness in improving seizure outcomes for patients with brain arteriovenous malformations, suggesting that interventional radiology can play a key role in epilepsy treatment [52]. In searching for predictive tools, Bronisz et al. identified serum proteins associated with blood–brain barrier function as potential biomarkers for seizure anticipation, offering a predictive edge in preventing seizure onset [53]. On a pharmacological note, Pieróg et al. investigated the effects of ellagic acid on seizure threshold, revealing its potential as an adjunct therapy in seizure management [54]. Maher et al. delved into the correlation between brain structure and function to unravel connectivity patterns preceding seizures, thus providing a blueprint for targeted therapeutic interventions [55]. Finally, the work of Arocha Pérez et al. focused on the semiology of seizures and cerebral perfusion patterns in drug-resistant focal epilepsies, contributing to a better understanding of neural network dynamics in epilepsy [56]. These insights together fortify our approach to epilepsy, encouraging a synthesis of interventional, preventative, pharmacological, and analytical research.

The asymmetry of the brain and skull in all dimensions makes it challenging to accurately measure the movement of the brain relative to the skull during complex acceleration or deceleration. Computational analysis, as shown in this study, is necessary for understanding these movements. Our work’s strength lies in applying SPH simulations in combination with high-order FEM to study CSF’s behavior during seizures. This method advanced our understanding of how force within the CSF is distributed and transmitted to the brain regions during seizures, essential for developing targeted interventions in neurology. This study reveals notable stress levels present in the brainstem, which can lead to symptoms such as dizziness or impaired motor function. In severe cases, paralysis, coma, or even death may occur [57]. Therefore, individuals who regularly experience seizures should take precautions by strengthening their neck area to safeguard against potential injuries to the brainstem.

The CSF surrounding our brain serves the important function of cushioning the brain from external blows. However, it is important to note that the CSF is primarily meant to protect against a single blow. This is because our bodies have evolved to protect the brain from simpler outside forces, such as being run over by an animal, being hit by primitive weapons like spears or clubs, or falling from a rock. In modern times, we often encounter more complex injuries, such as those resulting from automotive accidents. The CSF, unfortunately, lacks the ability to adequately protect our brains when we experience multiple episodes in quick succession, one after another. This is because the fluid surrounding our brain cannot keep up with the rapid changes in the velocity (i.e., acceleration and deceleration) of the skull when exposed to such complex loading conditions. Our bodies have evolved to withstand and protect against certain types of forces, but the increasing complexity of our modern environment has introduced new challenges. The CSF’s protective function, while effective against single blows, may not be sufficient to mitigate the effects of multiple traumatic events occurring in quick succession.

It is important to recognize these limitations and explore ways to enhance brain protection in situations involving complex loading conditions. By understanding the biomechanics of the brain and the limitations of the CSF, researchers and medical professionals can work towards developing improved strategies for brain protection and injury prevention in various scenarios, including automotive accidents and other high-impact situations. Hence, the CSF serves as a natural cushion for the brain, primarily protecting against single blows. The evolving nature of our environment and the introduction of more complex injuries necessitate further research and advancements in brain protection to address the limitations of the CSF in scenarios involving multiple traumatic events.

Our study has shed light on the complex physiological changes that occur in the brain during seizures, providing vital understanding of the mechanical interactions within the brain’s structural network. The simulations undertaken in this study demonstrated the crucial role of the CSF in mitigating potential brain damage during seizures. However, they also highlighted its limitations in protecting against multiple traumatic events occurring in quick succession. Interestingly, we noticed that during simulated seizures, the brain experienced significant contact pressure, effective stress, and shear stress, predominantly in the brainstem and posterior regions of the gyri. Furthermore, there was a significant backward relative movement of the brain to the skull, emphasizing the abrupt brain movement magnitude during a seizure. The leftward shift skewness of the brain is a key finding and needs further investigation to understand better its long-term neurological implications. Nonetheless, the generalizability of these findings may be constrained due to the simulation nature of this study.

The intricate changes underscore the need for robust protective mechanisms during high-impact scenarios like seizures. These findings indicate areas for improvement in existing seizure management strategies and give impetus to explore enhanced protective mechanisms during seizure episodes. This calls for further improvement of our understanding of brain biomechanics and developing better seizure management strategies. Further research could explore strategies for enhancing CSF protection; understanding metabolic changes due to increased energy consumption during seizures, the role of neurotransmitter imbalance, and its effects; and broadening the scope to include other types of seizures and demographics. Ultimately, with these insights and advancements, we foresee a beneficial impact on personalized neurology and neurosurgery. Our study accentuates the need for interdisciplinary research, combining inputs from neurology, biomechanics, and computational biology. This will afford a fundamentally sound landscape to devise therapeutic procedures and ensure healthy lives for individuals experiencing seizure episodes.

Considering recent studies that have underlined the importance of intracranial compliance as a valuable clinical and engineering measure for evaluating a wide range of brain disorders [58], it is suggested that the field would benefit from research that builds upon our current methodology. For future studies, we recommend replicating the procedures presented in our paper with a focused aim to investigate the sensitivity and impact of intracranial compliance on the assessment of parameters associated with seizures. By integrating intracranial compliance as a variable in computational simulations and experimental designs similar to those used in our study [59], researchers can gain more nuanced insights into how this compliance affects brain dynamics during seizure episodes. This approach will not only refine our understanding of the mechanical interactions within the brain’s structural network but can also inform the development of new diagnostics and treatment strategies that accurately account for intracranial compliance, further enhancing patient care for those with seizure disorders.

It is widely acknowledged among medical professionals that no two seizures are alike. Each seizure presents with unique characteristics, such as varying durations, the presence or absence of premonitory auras, and distinct post-seizure recovery times. Furthermore, patients may experience different types of seizures, sometimes even within the same day, further complicating the understanding of this complex phenomenon. Admittedly, it raises concerns regarding this model’s ability to accurately replicate the multifaceted nature of seizures. We do not claim to have captured the essence of all seizures; it is clear that this model does not adequately encompass the diverse range of seizure types described in the neurology literature. For example, the observed brain movements, which appear to push the brain backward, are not consistent across various seizure types, such as tonic–clonic, motor partial, sensory partial, and gelastic seizures. The findings and conclusions drawn from this study are specific to the particular case examined and are not generalizable to a larger population. The exact mechanisms underlying seizures in general remain unknown.

The simulations presented here have provided some initial insights into the biomechanical relationship between the brain and CSF during seizures. Notably, the transient increase in intracranial pressure and associated hemispheric swelling, phenomena characteristic of severe seizure episodes such as status epilepticus [60], represent critical factors that need to be integrated into the model. This would allow us to explore the buffering capabilities of CSF under such conditions. Although this model presents a step towards understanding the FSI during seizure events, we recognize that the complexity of these interactions requires further refinement of our simulations. By advancing our model to better align with the physiological realities observed in clinical settings, we aim to provide a more detailed representation of the CSF’s role as both a shock absorber during acute increases in intracranial pressure and a potential mitigator of brain tissue deformation. The insights gained from these improved simulations could be instrumental in enhancing patient-specific management strategies for those affected by seizure disorders.

Similarities in how the brain responds to different types of trauma—one caused by internal electrical disturbances and the other by external mechanical forces—can be identified by comparing this study on the effects of seizures on brain dynamics with one analyzing brain dynamics in, e.g., shaken baby syndrome [61]. Both the study of the impact of seizures on brain dynamics and a study analyzing brain dynamics in cases of abusive head trauma are concerned with the biomechanical responses and injuries that result from traumatic forces exerted on the brain. In the context of a seizure, stress and strain can result from the convulsions caused by abnormal electrical activity, whereas abusive head trauma involves direct physical forces causing rapid acceleration and deceleration of the head and brain. Both situations lead to complex physiological responses within the brain structure, including changes in CSF behavior, potential disruption of neural networks, and exertion of shear stress and contact pressure on brain tissues. Understanding the biomechanical effects in both scenarios is crucial for developing protective strategies, improving diagnostic techniques, and creating more effective treatment plans to mitigate long-term damage to the brain. While the causes of the brain dynamics in each case differ—one being internal and electrical, the other external and mechanical—the study of their impacts can yield insights into the brain’s response to different types of trauma and the role of CSF in protecting the brain under such stress. Research into these varied traumatic events is instrumental to understanding the resilience and vulnerability of the brain’s structure and functions. It allows for the creation of enhanced protective equipment, like helmets for military personnel when their brains are exposed to the intense pressures from military blasts [62,63], the refinement of child safety protocols to prevent abusive head trauma, and the advancement of medical interventions to better manage seizure disorders. The aim remains consistent across studies: to minimize injury and optimize outcomes for those who have suffered or are at risk of brain trauma.

## 5. Conclusions

This study presents valuable insights into the role of CSF in cushioning the brain during seizure-induced shaking episodes and highlights the bedrock of physiological changes occurring in different parts of the brain. Our investigation, using SPH-FEM simulations, offered a comprehensive view of how force is distributed within the CSF, thus affecting different brain areas. We found significant stress levels, contact pressure, and shear stress exerted on the brain during simulated seizures, with maximum prominence in the brainstem and posterior regions of the gyri. Moreover, our results indicate a noticeable movement of the brain relative to the skull during seizures, primarily towards the posterior side, highlighting the significance of abrupt brain movements during such events.

Furthermore, our analysis brought attention to the limitations of the CSF, which mostly protects the brain against single blows. In our evolving, complex environment, the increase in incidents involving multiple successive traumas, such as car accidents, necessitates rethinking ways to enhance brain protection. These findings underline the importance of improving our understanding of brain biomechanics, developing better seizure management strategies, and enhancing protective mechanisms during high-impact scenarios. More so, it emphasizes the potential of computational simulations in predicting the biophysiological responses of the brain, ultimately guiding the advancement of personalized neurology and neurosurgery interventions.

## Data Availability

Data are contained within the article.

## Abbreviations

The following abbreviations are used in this manuscript:

CSF	Cerebrospinal Fluid
DICOM	Digital Imaging and Communications in Medicine
A2AR	A2A Receptors
BOLD	Blood Oxygenation Level-Dependent
SPH	Smoothed Particle Hydrodynamics
FEM	Finite Element Method
FSI	Fluid–Structure Interaction

## References

[1] Kaculini C.M., Tate-Looney A.J., Seifi A. (2021). The History of Epilepsy: From Ancient Mystery to Modern Misconception. Cureus.

[2] Zack M.M., Kobau R. (2017). National and State Estimates of the Numbers of Adults and Children with Active Epilepsy—United States, 2015. MMWR. Morb. Mortal. Wkly. Rep..

[3] Moon H.J., Lee H., Yoon D., Koo Y.S., Shin J.Y., Lee S.Y. (2023). Premature Mortality and Causes of Death among People with Epilepsy: A Nationwide Population-Based Incident Cohort Study. Neurology.

[4] Berg A. (2001). Mortality in Epilepsy. Epilepsy Curr..

[5] Ghosh S., Sinha J.K., Ghosh S., Sharma H., Bhaskar R., Narayanan K.B. (2023). A Comprehensive Review of Emerging Trends and Innovative Therapies in Epilepsy Management. Brain Sci..

[6] Ostendorf A.P. (2023). Epilepsy Impacts Families and Communities: Persistent Gaps and Inequities. Semin. Pediatr. Neurol..

[7] Gavvala J.R., Schuele S.U. (2016). New-Onset Seizure in Adults and Adolescents. JAMA.

[8] Lehnertz K., Bialonski S., Horstmann M.T., Krug D., Rothkegel A., Staniek M., Wagner T. (2009). Synchronization phenomena in human epileptic brain networks. J. Neurosci. Methods.

[9] Shellhaas R.A. (2019). Seizure classification, etiology, and management. Handbook of Clinical Neurology.

[10] Holmes G.L. (2016). Effect of Seizures on the Developing Brain and Cognition. Semin. Pediatr. Neurol..

[11] Tóth K., Hofer K.T., Kandrács Á., Entz L., Bagó A., Erőss L., Jordán Z., Nagy G., Sólyom A., Fabó D. (2017). Hyperexcitability of the network contributes to synchronization processes in the human epileptic neocortex. J. Physiol..

[12] Dabrowska N., Joshi S., Williamson J., Lewczuk E., Lu Y., Oberoi S., Brodovskaya A., Kapur J. (2019). Parallel pathways of seizure generalization. Brain.

[13] Wenzel M., Hamm J.P., Peterka D.S., Yuste R. (2019). Acute Focal Seizures Start As Local Synchronizations of Neuronal Ensembles. J. Neurosci..

[14] During M., Spencer D. (1993). Extracellular hippocampal glutamate and spontaneous seizure in the conscious human brain. Lancet.

[15] Green A.R., Minchin M.C., Vincent N.D. (1987). Inhibition of GABA release from slices prepared from several brain regions of rats at various times following a convulsion. Br. J. Pharmacol..

[16] Hamer H.M. (2005). Motor cortex excitability in focal epilepsies not including the primary motor area—A TMS study. Brain.

[17] He X., Chaitanya G., Asma B., Caciagli L., Bassett D.S., Tracy J.I., Sperling M.R. (2019). Disrupted basal ganglia–thalamocortical loops in focal to bilateral tonic-clonic seizures. Brain.

[18] Lisgaras C.P., Scharfman H.E. (2022). Robust chronic convulsive seizures, high frequency oscillations, and human seizure onset patterns in an intrahippocampal kainic acid model in mice. Neurobiol. Dis..

[19] Nass R.D., Zur B., Elger C.E., Holdenrieder S., Surges R. (2019). Acute metabolic effects of tonic-clonic seizures. Epilepsia Open.

[20] Nersesyan H., Hyder F., Rothman D.L., Blumenfeld H. (2004). Dynamic fMRI and EEG Recordings during Spike-Wave Seizures and Generalized Tonic-Clonic Seizures in WAG/Rij Rats. J. Cereb. Blood Flow Metab..

[21] Gonçalves F.B., Garcia-Gomes M.S., Silva-Sampaio A.C., Kirsten T.B., Bondan E.F., Sandini T.M., Flório J.C., Lebrun I., de C. Coque A., Alexandre-Ribeiro S.R. (2023). Progressive tremor and motor impairment in seizure-prone mutant tremor mice are associated with neurotransmitter dysfunction. Behav. Brain Res..

[22] Tran L.V., Tran H.M., Le T.M., Huynh T.T.M., Tran H.T., Dao S.V.T. (2022). Application of Machine Learning in Epileptic Seizure Detection. Diagnostics.

[23] Ben-Ari Y. (2001). Cell Death and Synaptic Reorganizations Produced by Seizures. Epilepsia.

[24] Blumenfeld H., Varghese G.I., Purcaro M., Motelow J., Enev M., McNally K.A., Levin A., Hirsch L.J., Tikofsky R., Zubal I.G. (2009). Cortical and subcortical networks in human secondarily generalized tonic-clonic seizures. Brain.

[25] Brodovskaya A., Shiono S., Kapur J. (2021). Activation of the basal ganglia and indirect pathway neurons during frontal lobe seizures. Brain.

[26] Canas P.M., Porciúncula L.O., Simões A.P., Augusto E., Silva H.B., Machado N.J., Gonçalves N., Alfaro T.M., Gonçalves F.Q., Araújo I.M. (2018). Neuronal Adenosine A2A Receptors Are Critical Mediators of Neurodegeneration Triggered by Convulsions. eNeuro.

[27] DeSalvo M.N., Schridde U., Mishra A.M., Motelow J.E., Purcaro M.J., Danielson N., Bai X., Hyder F., Blumenfeld H. (2010). Focal BOLD fMRI changes in bicuculline-induced tonic-clonic seizures in the rat. NeuroImage.

[28] Ghadiri T., Gorji A., Vakilzadeh G., Hajali V., Khodagholi F., Sharifzadeh M. (2020). Neuronal injury and death following focal mild brain injury: The role of network excitability and seizure. Iran. J. Basic Med. Sci..

[29] Sakka L., Coll G., Chazal J. (2011). Anatomy and physiology of cerebrospinal fluid. Eur. Ann. Otorhinolaryngol. Head Neck Dis..

[30] Camfield C., Camfield P. (2015). Injuries from seizures are a serious, persistent problem in childhood onset epilepsy: A population-based study. Seizure.

[31] Lun M.P., Monuki E.S., Lehtinen M.K. (2015). Development and functions of the choroid plexus—Cerebrospinal fluid system. Nat. Rev. Neurosci..

[32] Vezzani A., Granata T. (2005). Brain Inflammation in Epilepsy: Experimental and Clinical Evidence. Epilepsia.

[33] Xu F., Wang J., Yang Y., Wang L., Dai Z., Han R. (2023). On methodology and application of smoothed particle hydrodynamics in fluid, solid and biomechanics. Acta Mech. Sin..

[34] Toma M., Chan-Akeley R., Arias J., Kurgansky G.D., Mao W. (2021). Fluid–Structure Interaction Analyses of Biological Systems Using Smoothed-Particle Hydrodynamics. Biology.

[35] Luo Y., Li Z., Chen H. (2012). Finite-element study of cerebrospinal fluid in mitigating closed head injuries. Proc. Inst. Mech. Eng. Part H J. Eng. Med..

[36] Mikhal J., Geurts B.J. (2012). Development and application of a volume penalization immersed boundary method for the computation of blood flow and shear stresses in cerebral vessels and aneurysms. J. Math. Biol..

[37] Zimmerman K.A., Cournoyer J., Lai H., Snider S.B., Fischer D., Kemp S., Karton C., Hoshizaki T.B., Ghajari M., Sharp D.J. (2022). The biomechanical signature of loss of consciousness: Computational modelling of elite athlete head injuries. Brain.

[38] Toma M., Nguyen P.D.H. (2019). Coup-contrecoup brain injury: Fluid–structure interaction simulations. Int. J. Crashworthiness.

[39] Fry F.J., Barger J.E. (1978). Acoustical properties of the human skull. J. Acoust. Soc. Am..

[40] Tyler W.J. (2012). The mechanobiology of brain function. Nat. Rev. Neurosci..

[41] Elkin B.S., Azeloglu E.U., Costa K.D., Morrison B. (2007). Mechanical Heterogeneity of the Rat Hippocampus Measured by Atomic Force Microscope Indentation. J. Neurotrauma.

[42] Gefen A., Gefen N., Zhu Q., Raghupathi R., Margulies S.S. (2003). Age-Dependent Changes in Material Properties of the Brain and Braincase of the Rat. J. Neurotrauma.

[43] Kruse S.A., Rose G.H., Glaser K.J., Manduca A., Felmlee J.P., Jack C.R., Ehman R.L. (2008). Magnetic resonance elastography of the brain. NeuroImage.

[44] Moore S.W., Sheetz M.P. (2011). Biophysics of substrate interaction: Influence on neural motility, differentiation, and repair. Dev. Neurobiol..

[45] Lui A.C.P., Polis T.Z., Cicutti N.J. (1998). Densities of cerebrospinal fluid and spinal anaesthetic solutions in surgical patients at body temperature. Can. J. Anaesth..

[46] Pellegrini L., Bonfio C., Chadwick J., Begum F., Skehel M., Lancaster M.A. (2020). Human CNS barrier-forming organoids with cerebrospinal fluid production. Science.

[47] Hiscox L.V., McGarry M.D.J., Schwarb H., Houten E.E.W.V., Pohlig R.T., Roberts N., Huesmann G.R., Burzynska A.Z., Sutton B.P., Hillman C.H. (2020). Standard-space atlas of the viscoelastic properties of the human brain. Hum. Brain Mapp..

[48] Toma M., Nguyen P.D. (2018). Fluid–structure interaction analysis of cerebrospinal fluid with a comprehensive head model subject to a rapid acceleration and deceleration. Brain Inj..

[49] Nahum A.M., Gatts J.D., Gadd C.W., Danforth J. (1968). Impact Tolerance of the Skull and Face. Proceedings of the 12th Stapp Car Crash Conference (1968).

[50] Rooks T.F., Chancey V.C., Baisden J.L., Yoganandan N. (2023). Regional Strain Response of an Anatomically Accurate Human Finite Element Head Model Under Frontal Versus Lateral Loading. Mil. Med..

[51] Yu C., Wang F., Wang B., Li G., Li F. (2020). A Computational Biomechanics Human Body Model Coupling Finite Element and Multibody Segments for Assessment of Head/Brain Injuries in Car-To-Pedestrian Collisions. Int. J. Environ. Res. Public Health.

[52] Nurimanov C., Babi A., Menlibayeva K., Makhambetov Y., Kaliyev A., Zholdybayeva E., Akshulakov S. (2022). Effect of Targeted Embolization on Seizure Outcomes in Patients with Brain Arteriovenous Malformations. Diagnostics.

[53] Bronisz E., Cudna A., Wierzbicka A., Kurkowska-Jastrzębska I. (2022). Serum Proteins Associated with Blood–Brain Barrier as Potential Biomarkers for Seizure Prediction. Int. J. Mol. Sci..

[54] Pieróg M., Socała K., Wyska E., Poleszak E., Wlaź P. (2021). Effect of Ellagic Acid on Seizure Threshold in Two Acute Seizure Tests in Mice. Molecules.

[55] Maher C., D’Souza A., Barnett M., Kavehei O., Wang C., Nikpour A. (2022). Structure-Function Coupling Reveals Seizure Onset Connectivity Patterns. Appl. Sci..

[56] Arocha Pérez J.L., Morales Chacón L.M., Batista García Ramo K., Galán García L. (2022). Sequential Semiology of Seizures and Brain Perfusion Patterns in Patients with Drug-Resistant Focal Epilepsies: A Perspective from Neural Networks. Behav. Sci..

[57] Benghanem S., Mazeraud A., Azabou E., Chhor V., Shinotsuka C.R., Claassen J., Rohaut B., Sharshar T. (2020). Brainstem dysfunction in critically ill patients. Crit. Care.

[58] Gholampour S., Yamini B., Droessler J., Frim D. (2022). A New Definition for Intracranial Compliance to Evaluate Adult Hydrocephalus after Shunting. Front. Bioeng. Biotechnol..

[59] Toma M., Kuo S.H. (2020). Computational Assessment of Risk of Subdural Hematoma Associated with Ventriculoperitoneal Shunt Placement. Computer Methods, Imaging and Visualization in Biomechanics and Biomedical Engineering.

[60] Shen J.Y., Saffari S.E., Yong L., Tan N.C.K., Tan Y.L. (2024). Evaluation of prognostic scores for status epilepticus in the neurology ICU: A retrospective study. J. Neurol. Sci..

[61] Toma M., Dehesa-Baeza A., Chan-Akaley R., Nguyen P.D.H., Zwibel H. (2020). Cerebrospinal Fluid Interaction with Cerebral Cortex during Pediatric Abusive Head Trauma. J. Pediatr. Neurol..

[62] Frankini E., Basile E.J., Syed F., Wei O.C., Toma M. (2023). Understanding Traumatic Brain Injuries in Military Personnel: Investigating the Dynamic Interplay of the Cerebrospinal Fluid and Brain During Blasts. Cureus.

[63] Syed F., Frankini E., Hurdle K., Garcia J., Chan-Akeley R., Toma M. (2023). Cushioning Effect of Conventional Padded Helmets on Interaction between Cerebrospinal Fluid and Brain after a Low-Speed Head Impact. Appl. Sci..

